# Supplementary material for: Mutations in PCYT2 disrupt etherlipid biosynthesis and cause a complex hereditary spastic paraplegia
Source: Brain. 2019 Oct 22;142(11):3382–97. doi: 10.1093/brain/awz291 (PMC6821184; doi:10.1093/brain/awz291)
Supplement: awz291_Supplementary_Data [file awz291_supplementary_data.zip › awz291-Suppl_data/Supplementary_Data4.zip!/Supplementary information 4 - plasma lipidomics.zip!/Plasma lipidomics/Control vs Patient1/Statistical plots and data/TG species/Volcano plot_alternative.pdf]

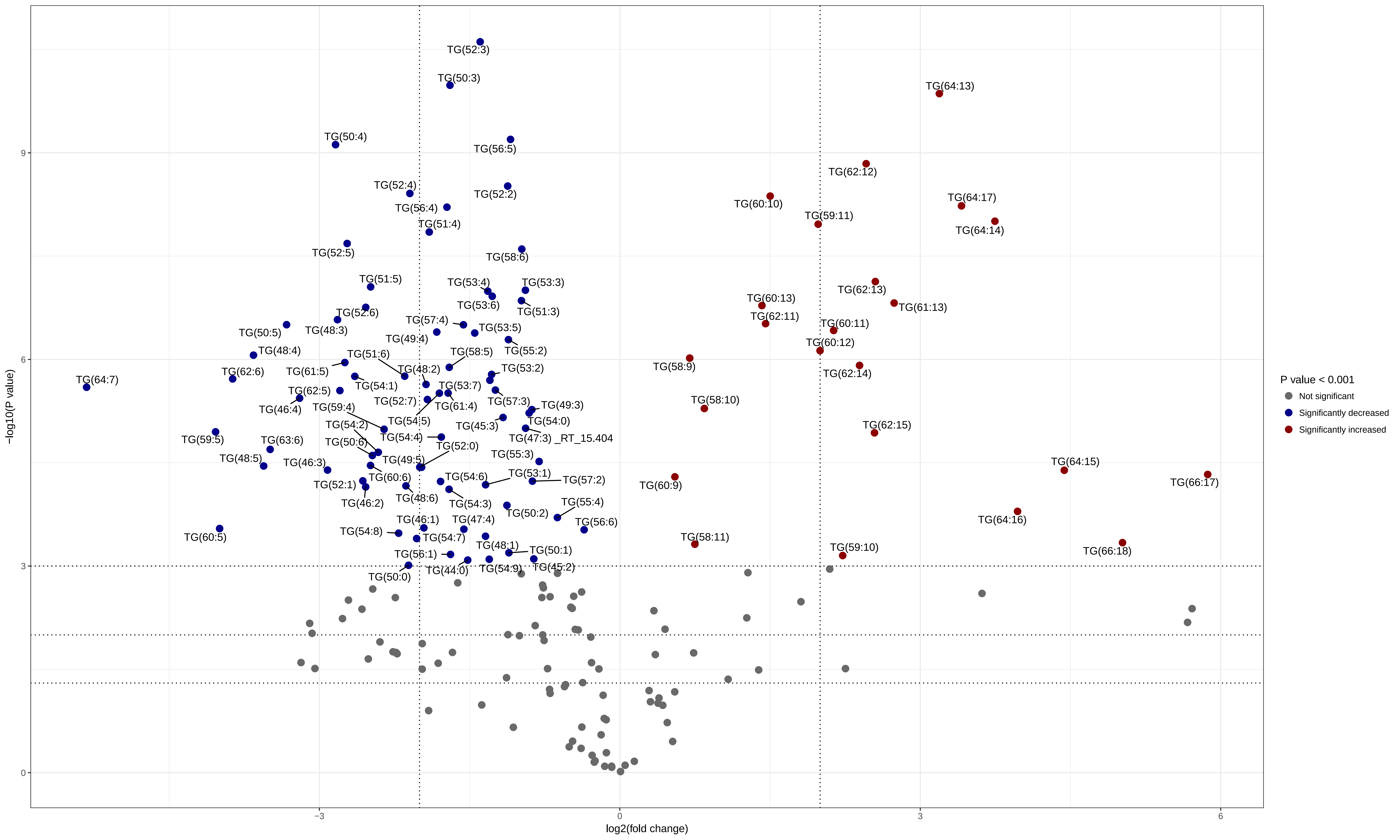

TG(52:3)
TG(50:3)
TG(64:13)
TG(50:4)
TG(56:5)
TG(62:12)
TG(52:4)
TG(52:2)
TG(60:10)
TG(64:17)
TG(56:4)
TG(59:11)
TG(51:4)
TG(64:14)
TG(52:5)
TG(58:6)
TG(51:5)
TG(53:4)
TG(53:3)
TG(62:13)
TG(60:13)
TG(61:13)
TG(53:6)
TG(51:3)
TG(52:6)
TG(62:11)
TG(57:4)
TG(60:11)
TG(50:5)
TG(48:3)
TG(53:5)
TG(49:4)
TG(60:12)
TG(48:4)
TG(58:5)
TG(55:2)
TG(51:6)
TG(58:9)
TG(62:14)
TG(64:7)
TG(62:6)
TG(61:5)
TG(48:2)
TG(53:2)
TG(54:1)
TG(53:7)
TG(62:5)
TG(52:7)
TG(57:3)
TG(58:10)
TG(46:4)
TG(59:4)
TG(61:4)
TG(49:3)
TG(54:5)
TG(54:0)
TG(54:2)
TG(45:3)
TG(62:15)
TG(59:5)
TG(63:6)
TG(50:6)
TG(54:4)
TG(47:3)_RT_15.404
TG(52:0)
TG(48:5)
TG(55:3)
TG(46:3)
TG(49:5)
TG(64:15)
TG(60:6)
TG(54:6)
TG(53:1)
TG(57:2)
TG(66:17)
TG(52:1)
TG(60:9)
TG(48:6)
TG(46:2)
TG(54:3)
TG(55:4)
TG(50:2)
TG(64:16)
TG(46:1)
TG(47:4)
TG(56:6)
TG(54:8)
TG(54:7)
TG(60:5)
TG(58:11)
TG(48:1)
TG(59:10)
TG(66:18)
TG(56:1)
TG(50:1)
TG(54:9)
TG(45:2)
TG(44:0)
TG(50:0)
9
6
3
0
−3
0
3
6
−log10(P value)
log2(fold change)
P value < 0.001
Not significant
Significantly decreased
Significantly increased
